# Mitochondrial gene heterogeneity of the bat soft tick *Argas vespertilionis* (Ixodida: Argasidae) in the Palaearctic

**DOI:** 10.1186/s13071-017-2037-4

**Published:** 2017-02-28

**Authors:** Sándor Hornok, Krisztina Szőke, Vuong Tan Tu, Jenő Kontschán, Nóra Takács, Attila D. Sándor, Ali Halajian, Gábor Földvári, Péter Estók, Olivier Plantard, Sara Epis, Tamás Görföl

**Affiliations:** 1 Department of Parasitology and Zoology, University of Veterinary Medicine, Budapest, Hungary; 2 Institute of Ecology and Biological Resources, Vietnam Academy of Science and Technology, Hanoi, Vietnam; 3 Plant Protection Institute, Centre for Agricultural Research, Hungarian Academy of Sciences, Budapest, Hungary; 4 Department of Parasitology and Parasitic Diseases, University of Agricultural Sciences and Veterinary Medicine, Cluj-Napoca, Romania; 5 Department of Biodiversity, School of Molecular and Life Sciences, Faculty of Science and Agriculture, University of Limpopo, Polokwane, South Africa; 6 Department of Zoology, Eszterházy Károly University, Eger, Hungary; 7 BIOEPAR, INRA, Oniris, Nantes, France; 8 Department of Biosciences, University of Milan, Milan, Italy; 9 Department of Zoology, Hungarian Natural History Museum, Budapest, Hungary

**Keywords:** Soft tick, Chiroptera, Phylogeny, Phylogeography, Cryptic species

## Abstract

**Background:**

Recently, a high degree of mitochondrial gene heterogeneity was demonstrated between conspecific ixodid ticks of bats in Eurasia. *Argas vespertilionis* is a soft tick species of mainly vespertilionid bats, also with a wide distribution in the Old World. The aim of this study was to investigate the morphology, mitochondrial gene heterogeneity and host range of *A. vespertilionis* in the Old World.

**Results:**

Altogether 318 soft tick larvae were collected from 17 bat species (belonging to six genera) in seven countries. Based on the general morphology (setal arrangement) of 314 *A. vespertilionis* larvae, and the detailed measurements of fifteen larvae, only minor morphological differences (in dorsal plate size and the type of serrate setae) were observed between specimens from Europe and Vietnam. On the other hand, cytochrome *c* oxidase subunit 1 (*cox*1) and 16S rRNA gene sequence analyses of 17 specimens showed that *A. vespertilionis* from Europe is genetically different (with up to 7.5% *cox*1 and 5.7% 16S rRNA gene sequence divergence) from specimens collected in Vietnam, and their phylogenetic separation is well supported.

**Conclusion:**

In its evaluated geographical range, no larval phenotypic differences justify the existence of separate species under the name *A. vespertilionis*. However, phylogenetic analyses based on two mitochondrial markers suggest that it represents a complex of at least two putative cryptic species. The broad host range of *A. vespertilionis* might partly explain its lower degree of mitochondrial gene heterogeneity in comparison with ixodid bat tick species over the same geographical region of Eurasia.

## Background

Soft ticks (Acari: Ixodida: Argasidae) include approximately 200 species that are obligate haematophagous ectoparasites of hosts from all classes of terrestrial vertebrates. *Argas vespertilionis* is a cosmopolitan soft tick species of mainly vespertilionid bats (especially *Pipistrellus* spp.), with a wide distribution in the Old World, from the Palaearctic to South Africa [1]. Apart from bats, *A. vespertilionis* was reported to feed on humans [1, 2] and domestic animals [3]. This soft tick species is a potential vector of zoonotic viruses [3] and bacteria [4], and the piroplasm *Babesia vesperuginis* [5].

The taxonomy of the Argasidae is controversial, because the majority of soft tick species can be assigned to more than one genus [6]. Accordingly, the taxonomical status of *A. vespertilionis* also appears to be uncertain. It was originally the type species of the genus *Carios* [7], but in the most recent list of valid tick names it is mentioned as a member of the genus *Argas* [8]. Recent phylogenetic analyses do not support *A. vespertilionis* as a member of the genus *Argas*, as this species has been misplaced into the subfamily Argasinae [9], and based on its 12S rRNA gene it should belong to Ornithodorinae [6]. In addition, the homogeneity of *A. vespertilionis* on the species level has long been questioned [7].

Recently, high degree of mitochondrial gene heterogeneity was demonstrated between conspecific ixodid ticks of bats in Eurasia [10]. The aim of this study was to investigate *A. vespertilionis* in the same context, i.e. its morphology, mitochondrial gene heterogeneity and host range in the Old World.

To date, molecular analyses of soft ticks focused on the 16S rRNA gene, which has the potential for resolving phylogenetic relationships among closely related species in Argasidae [6]. In addition to this gene, the 5’ region of the mitochondrial cytochrome *c* oxidase subunit 1 (*cox*1) gene was chosen for phylogenetic analysis of *A. vespertilionis* in the present study, because it is regarded as the standard marker for tick species identification by DNA barcoding [11], and is particularly suitable to track separation among soft tick species [12]. Since there are few soft tick *cox*1 reference sequences in GenBank, a South African isolate of *A. transgariepinus* and a neotropical isolate of an *Ornithodoros* sp. were also included in the *cox*1 phylogenetic analysis. The nomenclature used in the manuscript complies with the valid tick names listed by Guglielmone et al. [8], who follow Hoogstraal in his classification of the Argasidae, although that classification, including the re-assignment of *Carios* as a subgenus of *Argas*, has never been supported by any analysis using actual data.

## Methods

### Sample collection and morphological analyses

Soft ticks were collected from bats captured for ringing and monitoring purposes (Table 1). All ticks were stored in 70% ethanol. Morphological identification was based on the description of *A. vespertilionis* and *A. transgariepinus* larvae by Hoogstraal [7, 13], and of *Ornithodoros* larvae (on the genus level) according to Barros-Battesti et al. [14], and Jones & Clifford [15]. Structures of representative specimens from each country (*A. vespertilionis*: eight larvae from Vietnam, three larvae from Italy, four larvae from Romania; except *A. vespertilionis* from Kenya, which was damaged) were measured under a Jenaval light microscope (Carl Zeiss GmbH, Jena, Germany) after clearance with lactic acid. The means of these data sets were compared by using two-tailed Student’s *t*-test, and were considered significantly different if *P* < 0.05.

**Table 1:** Host species, place of collection and GenBank accession numbers for sequences from soft ticks used in this study

Species	Stage (n)	Host species	Country (Locality)	*cox*1	16S rRNA
*Argas vespertilionis*	larva (58)	*Pipistrellus pygmaeus*	Hungary (Mezőföld)	KX431953	KX831484
	larva (5)	*Myotis alcathoe*	Hungary (Bakony)	KX431955	KX831486
	larva (12)	*Eptesicus serotinus*	Hungary (Béda)	–	–
	larva (15)	*Pipistrellus pygmaeus*	Hungary (Dráva)	–	–
	larva (1)	*Pipistrellus kuhlii*	Hungary (Dráva)	–	–
	larva (27)	*Plecotus austriacus*	Hungary (Dráva)	KX431954	KX831485
	larva (6)	*Myotis dasycneme*	Hungary (Gemenc)	–	–
	larva (58)	*Pipistrellus nathusii*	Hungary (Gemenc)	–	–
	larva (10)	*Pipistrellus pygmaeus*	Hungary (Gemenc)	–	–
	larva (1)	*Pipistrellus pipistrellus*	Hungary (Kecső)	KX431954	KX831489
	larva (2)	*Eptesicus serotinus*	Hungary (Mecsek)	–	–
	larva (1)	*Myotis alcathoe*	Hungary (Mecsek)	–	–
	larva (18)	*Myotis brandtii*	Hungary (Mecsek)	–	–
	larva (27)	*Myotis dasycneme*	Hungary (Mecsek)	–	–
	larva (4)	*Nyctalus noctula*	Hungary (Mecsek)	–	–
	larva (1)	*Plecotus auritus*	Hungary (Mecsek)	–	–
	larva (5)	*Pipistrellus pipistrellus*	Hungary (Mecsek)	–	–
	larva (1)	*Pipistrellus pygmaeus*	Hungary (Mecsek)	–	–
	larva (19)	*Vespertilio murinus*	Hungary (Miskolc)	–	–
	larva (1)	*Myotis alcathoe*	Hungary (Nagyvisnyó)	–	–
	larva (1)	*Pipistrellus pipistrellus*	Hungary (Nagyvisnyó)	–	–
	larva (1)	*Pipistrellus pipistrellus*	Hungary (Noszvaj)	–	–
	larva (1)	*Pipistrellus pipistrellus*	Hungary (Ócsa)	KX431953	KX831488
	larva (2)	*Vespertilio murinus*	Hungary (Sopron)	KX431953	KX831487
	larva (2)	*Eptesicus serotinus*	Romania (Somova)	KX431954	KX831490
	larva (9)	*Pipistrellus pipistrellus*	Romania (Salciua)	–	–
	larva (6)	*Pipistrellus pipistrellus*	Italy (Bergamo)	KX431953–KX431954	KX831496–KX831498
	larva (7)	*Pipistrellus javanicus*	Vietnam (Can Gio)	KX431957	KX831492
	larva (3)	*Pipistrellus cf. abramus*	Vietnam (Thanh Hoa)	KX431958	KX831493
	larva (9)	*Pipistrellus cf. abramus*	Vietnam (Bach Long Vi)	KX431959–KX431960	KX831494–KX831495
	larva (1)	*Pipistrellus cf. rueppellii*	Kenya (South Horr)	KX431956	KX831491
*Argas transgariepinus*	larva (1)	*Pipistrellus hesperidus*	South Africa (Makhado)	KX431961	–
*Ornithodoros* sp.	larva (3)	*Balantiopteryx plicata*	Mexico (Chiapas)	KX431962	KX831499

### DNA extraction, molecular and phylogenetic analyses

DNA was extracted from the larvae with the QIAamp DNA Mini Kit (Qiagen, Hilden, Germany) according to the manufacturer’s instruction, including an overnight digestion in tissue lysis buffer with 6.6% Proteinase-K at 56 °C. Twenty DNA extracts were used in this study for molecular analyses (eight from Hungary, two from Romania, three from Italy, four from Vietnam, one from Kenya, one from South Africa and one from Mexico). From these samples two mitochondrial markers were amplified: a 710 bp long fragment of the cytochrome *c* oxidase subunit 1 (*cox*1) gene, and an approx. 460 bp part of the 16S rRNA gene, as reported [16].

PCR products were visualized in 1.5% agarose gel. Purification and Sanger dideoxy sequencing (twice for each sample) were done by Biomi Inc. (Gödöllő, Hungary). Obtained sequences were manually edited, then aligned and compared to reference GenBank sequences by nucleotide BLASTN program (https://blast.ncbi.nlm.nih.gov). Representative sequences were submitted to GenBank (see accession numbers in Table 1). The MEGA model selection method was applied to choose the appropriate model for phylogenetic analyses. In the phylogenetic analyses reference sequences with high coverage (i.e. 98–100% of the region amplified here) were retrieved from GenBank, and trimmed to the same start and stop positions (*cox*1: 652 bp in length, 16S rRNA gene: 439–442 bp in length). This dataset was resampled 1,000 times to generate bootstrap values. Phylogenetic analyses were conducted with the Maximum Likelihood method and Tamura-Nei model by using MEGA version 6.0.

## Results

Altogether 318 soft tick larvae were collected from 17 bat species (belonging to six genera) in seven countries. All, except four soft tick larvae, were morphologically identified as *A. vespertilionis* (Table 1). The majority of *A. vespertilionis* larvae (59.1%: 188 out of 318, CI: 53.5–64.6%) were found on *Pipistrellus* spp. (Table 1). *Myotis alcathoe* is a new host for this soft tick species.

One specimen from South Africa was identified as *A. transgariepinus* (based on idiosomal setae, palpal articles, coxae and tarsus I). Three larvae from Mexico represented the genus *Ornithodoros* (based on the elongated piriform dorsal plate with non-parallel sides)*,* but could not be identified on the species level because of the lack of hypostome.

**Fig. 1:**
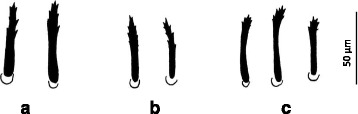
Schematic drawings of differences between serrate setae of *Argas vespertilionis* larvae from Romania (**a**), Italy (**b**) and Vietnam (**c**)

Measurements of selected, diagnostically important structures of *A. vespertilionis* larvae revealed no significant differences between specimens from Europe and Vietnam (Table 2), except for the length and width of the dorsal plate (plate length of ticks from Italy/Romania *vs* Vietnam: *t* = 3.49, *df* = 13, *P* = 0.004; plate width of ticks from Italy/Romania *vs* Vietnam: *t* = 3.21, *df* = 13, *P* = 0.006). Dorsal plate shape index (length:width ratio), as well as hypostome shape and dentition (4/4 anteriorly, 2/2 behind apex) were not significantly different between these categories. Sternal and anal setae were consistently pointed (needle-like), whereas dorsal setae were serrate. The morphology of serrate setae showed minor difference between geographically distant specimens (Fig. 1): larvae from Europe had separated surface protrusions in the upper half of setae, but those from Vietnam had grouped (tuft-like) fragmentation of the setal end.

**Table 2:** Measurements, i.e. size range (mean value) of selected structures with diagnostic importance in the case of *Argas vespertilionis* larvae from three geographical regions

		Italy (*n* = 3)	Romania (*n* = 4)	Vietnam (*n* = 8)
Idiosoma dorsum	Anterolateral setae (4th)	35–44 (38.3)	31–44 (37.8)	35–48 (42.3)
	Central setae (3rd)	31–45 (37.8)	38–41 (39.5)	33–41 (37.6)
	Posterolateral setae (4th)	51–61 (56.8)	55–65 (61.8)	56–71 (64)
	Dorsal plate length	200–208 (203.3)^a^	194–211 (204.8)^a^	193–201 (196.3)^b^
	Dorsal plate width	100–111 (106.3)^a^	101–113 (108.3)^a^	99–105 (101)^b^
	Dorsal plate ratio length:width	1.85–2 (1.92)	1.87–1.92 (1.89)	1.91–1.98 (1.94)
Idiosoma venter	Sternal setae (3rd)	25–27 (25.8)	23–30 (27.8)	20–35 (25.8)
	Circumanal setae (1st)	30–31 (30.3)	28–30 (29.3)	30–33 (30.8)
	Circumanal setae (2nd)	35–36 (35.3)	34–35 (34.8)	33–37 (35)
	Anal valve setae	32–38 (35)	32–38 (34.8)	35–39 (37)
	Posteromedian setae	23–29 (25.8)	25–28 (27)	25–30 (27.5)
Capitulum	Post-hypostomal setae	10–18 (13.8)	11–14 (12.3)	10–15 (11.9)
	Palpal length	165–180 (173.3)	174–176 (175)	165–176 (170.3)
	Hypostome length*	125–130 (127.5)	–	124–125 (124.5)
	Hypostome width (anterior)*	30–31 (30.5)	–	31–35 (33)
	Hypostome width (posterior)*	36–39 (37.5)	–	39–40 (39.5)
Legs	Tarsus I length	125–135 (129)	124–139 (128.3)	130–137 (132.8)
	Longest seta of tarsus I (near Haller’s organ)	36–45 (42.5)	43–47 (44.8)	38–55 (46.5)

Lengths are provided in μm, rounded to decimals (except for dorsal plate ratio). Values within a row having different superscript letters are significantly different

*Most larvae had broken hypostome; only two specimens from Italy and two from Vietnam allowed measurements

**Fig. 2:**
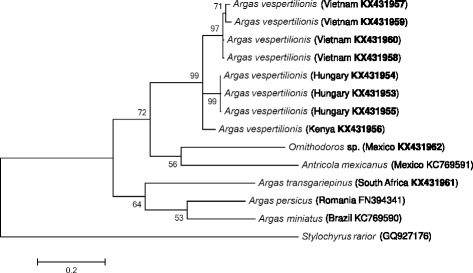
Phylogenetic relationships of *Argas vespertilionis* (collected in Hungary, Kenya and Vietnam) and other soft tick species, based on the cytochrome *c* oxidase subunit 1 (*cox*1) gene. *Cox*1 sequences of *A. vespertilionis* from Romania and Italy were identical with those from Hungary, therefore are not shown. Accession numbers of sequences from this study are highlighted in bold. Branch lengths represent the number of substitutions per site inferred according to the scale shown

Sequencing of the *cox*1 gene fragment was successful in the case of 17 samples (Table 1; Fig. 2). *Argas vespertilionis cox*1 sequences showed 0–2 nucleotide (0–0.3%) differences, i.e. 99.7–100% (650–652/652 bp) similarity between isolates from Hungary, Romania and Italy. Haplotypes from Europe had 37–38 nucleotide (5.7–5.8%) differences from an *A. vespertilionis* larva collected in Kenya, meaning 94.2–94.3% (614–615/652 bp) similarity with the latter. There was a more pronounced sequence divergence between specimens of *A. vespertilionis* from Europe and Vietnam, amounting to 46–49 nucleotide (7.1–7.5%) differences, i.e. only 92.5–92.9% (603–606/652 bp) similarity. The *cox*1 sequences of *A. vespertilionis* from Vietnam had 2–15 nucleotide (0.3–2.3%) differences from each other, amounting to 97.7–99.7% (637–650/652 bp) similarity, i.e. were more heterogeneous within Vietnam than between samples from three European countries. The topology of the *cox*1 phylogenetic tree reflected the above differences (with high support of separation of *A. vespertilionis* haplotypes both within Vietnam, and between Hungary and Vietnam). Clustering of *A. vespertilionis* isolates with two members of Ornithodorinae received moderate (72%) support (Fig. 2).

**Fig. 3:**
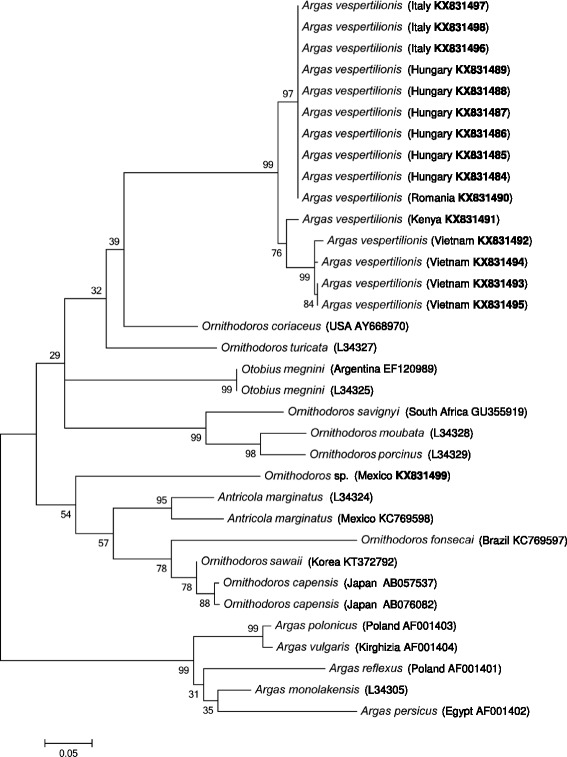
Phylogenetic relationships of *Argas vespertilionis* (collected in five countries), and other soft tick species, based on the 16S rRNA gene. Accession numbers of sequences from this study are highlighted in bold. Branch lengths represent the number of substitutions per site inferred according to the scale shown

Concerning the amplified part of the 16S rRNA gene, this was successfully sequenced from 16 samples. *Argas vespertilionis* had up to four nucleotide (0.9%) differences (437/441 bp = 99.1% similarity) between European haplotypes, whereas these had 20 nucleotide (4.5%) differences from the *A. vespertilionis* larva collected in Kenya (420/440 bp = 95.5% similarity), and 25 nucleotide (5.7%) differences (416/441 bp = 94.3% similarity) from *A. vespertilionis* larvae from Vietnam. The 16S rRNA gene sequences of *A. vespertilionis* from Vietnam had up to six nucleotide (1.4%) differences from each other, i.e. 98.6% (436/442 bp) similarity. Based on the 16S rRNA phylogenetic tree (Fig. 3), the separation of *A. vespertilionis* from Europe *vs* Kenya/Vietnam was highly supported (99%); *A. vespertilionis* was placed outside Argasinae, but its relationships among Ornithodorinae were only weakly supported (Fig. 3).

## Discussion

In this study 314 *A. vespertilionis* larvae were collected and morphologically investigated. Finding of only larvae of soft ticks on bats is in line with the life cycle of *A. vespertilionis*, i.e. larvae (unlike nymphs and adults) suck blood for several weeks on their bat hosts (14–31 days: [1]), therefore almost exclusively these can be collected from bats. In the present study *A. vespertilionis* was found on 15 bat species, most of which are already reported hosts (including four *Pipistrellus* spp., seven *Myotis* spp. and two *Eptesicus*, as well as two *Nyctalus* spp. [17, 18]).

While *A. vespertilionis* is mentioned in the most recent list of valid tick names as a member of the genus *Argas* [8], morphological and phylogenetic analyses do not support this assumption [6, 9]. Instead, based on its 12S rRNA gene, *A. vespertilionis* was demonstrated to belong to Ornithodorinae [6]. Phylogenetic analyses of the present study also reflected that haplotypes of *A. vespertilionis* clustered outside the Argasinae.

During the past few decades scientific debate tried to establish morphological features suitable to solve the taxonomical uncertainty among the Argasidae. In the larval stage the number of setae according to anatomical location is an important feature to recognize genera [14], and the shape of dorsal plate, the morphology of hypostome and the length of setae may be used to distinguish closely related species [15, 19].

The possibility that more than one species exist under the name *A. vespertilionis* has already been suggested by Hoogstraal [7], but this remained hitherto unevaluated. In this study the great majority of relevant parameters were not significantly different between *A. vespertilionis* larvae from Europe and Vietnam, although these larvae proved to be well separated based on two mitochondrial genetic markers. Similarly, in a previous study comparing neotropical bat soft ticks, selected measurements (length of certain setae) differed slightly between larvae from different countries, but these were considered to represent the same species [19]. Intraspecific variations in body outline of *A. vespertilionis* have also been reported [7]. Furthermore, despite the differences in the mean length and width of the dorsal plate between *A. vespertilionis* from Europe and Vietnam, as demonstrated here, these alone cannot serve to delineate species, because the shape of the dorsal plate (reflected here by similar length:width ratios) is regarded as more relevant in this context [15, 19].

A minor difference was also observed between the serrate setae of *A. vespertilionis* larvae collected in distant regions of Eurasia. However, while the types of fringed setae were reported to be different between larvae of closely related *Ornithodoros* spp. [15], the latter were also shown to differ in the ranges of their setal lengths and hypostome (unlike *A. vespertilionis* larvae here). Therefore, in the absence of further distinguishing characteristics, the present data suggest that *A. vespertilionis* in Europe and Vietnam belong to the same species, and observed minor differences (i.e. dorsal plate size) should be interpreted as intraspecific variations between populations. The morphology of specimens analyzed in this study also suggests that they are conspecific with *A. vespertilionis* reported from Japan [20].

On the other hand, specimens from Europe and Vietnam had *cox*1 sequence divergence (7.1–7.5%) exceeding that proposed for closely related ixodid tick species (6%, see [11]). Accordingly, morphologically similar, but genetically distinct populations of *A. vespertilionis* exist in Europe and Southeast Asia, suggesting that this soft tick should be regarded as a complex (group) of at least two putative cryptic species. This seems to be justified from the morphology of the larval stage alone (because differences between argasid larvae served to describe new soft tick species, e.g. in [15]), but morphological investigation of adult specimens from both regions and molecular/phylogenetic analyses of nuclear markers (18S and 28S rRNA genes) should ultimately confirm this conclusion.

Compared in the same context, the sequence divergence between *A. vespertilionis* from Kenya and Europe was less pronounced than between samples from Europe and Vietnam, suggesting that genetic exchange has been more likely in this direction (although a larger sample size from sub-Saharan Africa is needed to draw final conclusion in this respect). In support of this possibility, some of the main hosts of *A. vespertilionis* in the present study, most notably *Pipistrellus nathusii* is known to migrate long distances (up to 1,900 km) in the north-eastern to south-western direction [21]. Another important host, *P. kuhlii* is widespread in certain regions across Europe, the Middle-East, North Africa and Asia [22].

In Eurasia, high degree of mitochondrial gene heterogeneity (i.e. up to 16% *cox*1 sequence divergence) has recently been demonstrated between ixodid bat ticks that had been regarded as conspecific [10]. This was explained by the preference of each tick species for bat hosts from a single genus, as well as by the geographical separation of relevant bat host species [10]. In comparison with ixodid bat ticks, the less pronounced difference (in terms of both morphology and genetics) between geographically distant isolates of *A. vespertilionis*, as shown here, may root in the fact that this soft tick species has a broad host spectrum (involving vespertilionid bats from at least six genera, as also shown here), thus preventing complete allopatric separation of its populations.

## Conclusions

In its evaluated geographical range, no larval phenotypic differences justify the existence of separate species under the name *A. vespertilionis*. However, phylogenetic analyses based on two mitochondrial markers suggest that it represents a complex of at least two putative cryptic species. The broad host range of *A. vespertilionis* might partly explain its lower degree of mitochondrial gene heterogeneity in comparison with ixodid bat tick species over the same geographical region of Eurasia.

## Abbreviations

*cox*1: Cytochrome *c* oxidase subunit 1
df: Degrees of freedom
rRNA: Ribosomal ribonucleic acid

## References

[1] Hoogstraal H (1956). Argas vespertilionis. African Ixodoidea I. Ticks of the Sudan.

[2] Jaenson TG, Tälleklint L, Lundqvist L, Olsen B, Chirico J, Mejlon H (1994). Geographical distribution, host associations, and vector roles of ticks (Acari: Ixodidae, Argasidae) in Sweden. J Med Entomol.

[3] Manzano-Román R, Díaz-Martín V, de la Fuente J, Pérez-Sánchez R. Soft ticks as pathogen vectors: distribution, surveillance and control. In: Manjur Shah M (ed.) Parasitology. 2012. http://www.intechopen.com/. [Accessed 23 June 2016].

[4] Socolovschi C, Kernif T, Raoult D, Parola P (2012). *Borrelia*, *Rickettsia*, and *Ehrlichia* species in bat ticks, France, 2010. Emerg Infect Dis.

[5] Gardner RA, Molyneux DH (1987). *Babesia vesperuginis*: natural and experimental infections in British bats (Microchiroptera). Parasitology.

[6] Burger TD, Shao R, Labruna MB, Barker SC (2014). Molecular phylogeny of soft ticks (Ixodida*:* Argasidae) inferred from mitochondrial genome and nuclear rRNA sequences. Ticks Tick Borne Dis.

[7] Hoogstraal H (1958). Bat ticks of the genus *Argas* (Ixodoidea, Argasidae). 3. The subgenus *Carios*. A redescription of *A.* (*C.*) *vespertilionis* (Latreille, 1802), and variation within an Egyptian population. Ann Ent Soc Am.

[8] Guglielmone AA, Robbins RG, Apanaskevich DA, Petney TN, Estrada-Pena A, Horak IG (2010). The Argasidae, Ixodidae and Nuttalliellidae (Acari: Ixodida) of the world: a list of valid species names. Zootaxa.

[9] Klompen JSH, Oliver JH (1993). Systematic relationships in the soft ticks (Acari: Ixodida: Argasidae). Syst Entomol.

[10] Hornok S, Estrada-Peña A, Kontschán J, Plantard O, Kunz B, Mihalca AD, et al. High degree of mitochondrial gene heterogeneity in the bat tick species *Ixodes vespertilionis*, *I. ariadnae* and *I. simplex *from Eurasia. Parasit Vectors. 2015;8:457. 10.1186/s13071-015-1056-2 PMC4573304 26382218

[11] Lv J, Wu S, Zhang Y, Chen Y, Feng C, Yuan X (2014). Assessment of four DNA fragments (COI, 16S rDNA, ITS2, 12S rDNA) for species identification of the Ixodida (Acari: Ixodida). Parasit Vectors.

[12] Cruikshank RH (2002). Molecular markers for the phylogenetics of mites and ticks. Exp Appl Acarol.

[13] Hoogstraal H (1957). Bat Ticks of the genus *Argas* (Ixodoidea, Argasidae) 2. *Secretargas* new subgenus and *A. transgariepinus* White, 1846, its adult and immature stages; with a definition of the subgenus *Argas*. Ann Ent Soc Am.

[14] Barros-Battesti DM, Ramirez DG, Landulfo GA, Faccini JHL, Dantas-Torres F, Labruna MB (2013). Immature argasid ticks: diagnosis and keys for Neotropical region. Rev Bras Parasitol Vet.

[15] Jones EK, Clifford CM. The systematics of the subfamily Ornithodorinae (Acarina: Argasidae). V. A revised key to larval Argasidae of the western hemisphere and description of seven new species of *Ornithodoros*. Ann Ent Soc Am. 1972;65:730–40.

[16] Hornok S, Takács N, Szőke K, Kunz B (2015). First record of *Ixodes ariadnae* in Germany. Acta Vet Hung.

[17] Siuda K, Stanko M, Piksa K, Gorz A (2009). Ticks (Acari: Ixodida) parasitizing bats in Poland and Slovakia. Wiad Parazytol.

[18] Frank R, Kuhn T, Werblow A, Liston A, Kochmann J, Klimpel S (2015). Parasite diversity of European *Myotis* species with special emphasis on *Myotis myotis* (Microchiroptera, Vespertilionidae) from a typical nursery roost. Parasit Vectors.

[19] Venzal JM, Nava S, Terassini FA, Orgrsewalska M, Camargo LMA, Labruna MB (2013). *Orntihodoros peropteryx* (Acari: Argasidae) in Bolivia: an argasid tick with a single nymphal stage. Exp Appl Acarol.

[20] Yamaguti N, Tipton VJ, Keegan HL, Toshioka S (1971). Ticks in Japan, Korea and Ryukyu islands. Brigham Young Univ Sci Bull Biol Ser.

[21] Hutterer R, Ivanova T, Meyer-Cords C, Rodrigues L (2005). Bat migrations in Europe. A review of banding data and literature.

[22] Bray TC, Mohammed OB, Alagaili AN (2013). Phylogenetic and demographic insights into Kuhl’s Pipistrelle, *Pipistrellus kuhlii*, in the Middle East. PLoS One.

